# Heart Failure, Kidney Function, and Elderly Age, Rather than Levofloxacin Therapy, Are Associated with QTc Prolongation in COVID-19 Patients

**DOI:** 10.3390/jcm14114006

**Published:** 2025-06-05

**Authors:** Katarzyna Wilk-Śledziewska, Rafał Śledziewski, Małgorzata Gryciuk, Piotr Jan Sielatycki, Aleksandra Zbroch, Franciszek Kukliński, Edyta Zbroch

**Affiliations:** 1 Department of Internal Medicine and Hypertension, Medical University of Bialystok, Zurawia 14, 15-540 Bialystok, Poland; mgryciuk1@gmail.com (M.G.); piotr.sielatycki@gmail.com (P.J.S.); olaz@onet.pl (A.Z.); franciszek.kulkinski@gmail.com (F.K.); edyta.zbroch@umb.edu.pl (E.Z.); 2 Department of Radiology, Medical University of Bialystok, 15-276 Bialystok, Poland; rafal.sledziewski@umb.edu.pl

**Keywords:** SARS-CoV-2, cardiovascular risk factors, hypertension, heart failure, creatinine, QTc prolongation, arrhythmias

## Abstract

**Background:** Prolongation of the QT interval is directly related to the risk of ventricular arrhythmias and sudden cardiac death. Age, comorbidities, and treatment schemes have been shown to influence its prolongation and may also significantly affect the course of SARS-CoV-2 infection. Fluoroquinolones, widely used during the COVID-19 pandemic, are known for their ability to prolong the QT interval. Risk of ventricular arrhythmias has also been reported in patients with infectious diseases, and this risk may have been associated with high levels of interleukin-6 (IL-6). **Purpose:** The aim of this study is to evaluate the effect of levofloxacin on the corrected QT interval in patients with COVID-19, as well as to identify sociodemographic, clinical, and biochemical parameters associated with QTc interval prolongation among patients with COVID-19. **Patients and Methods:** The medical records of 93 patients hospitalized for COVID-19 were retrospectively analyzed, focusing on the presence of comorbidities and treatment with levofloxacin. Selected sociodemographic, clinical, and biochemical parameters were then statistically analyzed, with emphasis on their effect on the corrected QTc interval. The QTc interval was calculated according to the Bazett formula. **Results:** Levofloxacin use was not significantly associated with QTc interval. Statistical analysis identified creatinine, heart failure and atrial fibrillation as significant predictors of QTc interval prolongation. The trends towards QTc interval prolongation observed with hypokalaemia and hypertension suggest that these factors may also contribute to QTc interval variability and should be taken into account when assessing arrhythmia risk. **Conclusions:** Our retrospective study indicates that QTc prolongation results from the interplay of multiple factors.

## 1. Introduction

### The Novel Coronavirus and QTc Prolongation

The novel acute respiratory syndrome coronavirus (SARS-CoV-2), responsible for the coronavirus pandemic that started in 2019, affects the health of infected patients in a manner dependent on pre-existing comorbidities [1]. COVID-19 patients with hypertension, congestive heart failure, diabetes, chronic kidney disease, or cancer have a higher risk of severe COVID-19 infection [2]. It can be assumed that older patients hospitalized for a viral infection, have a higher potential risk of death due to the presence of cardiovascular risk factors and other comorbidities [3]. To date, the exact cause of increased mortality due to COVID-19 in patients with multimorbidity has not been clearly assessed. Both multimorbidity and age of patients influence the risk of death from cardiovascular complications. One of the main and most well-studied risk factors for fatal ventricular arrhythmias and sudden cardiac death is QT interval prolongation. The mechanism of QT interval prolongation is multifactorial and includes cardiomyocyte hypertrophy, increased left ventricular mass, and consequent changes in left ventricular repolarization.

Patients with chronic kidney disease (CKD) are particularly susceptible to ventricular arrhythmias—the leading cause of death in dialysis patients [4]. The pathophysiology of arrhythmias in this population is complex and seems to be associated with water and electrolyte disorders, hormonal conditions and the dialysis procedure itself. Admittedly, little is known about the clinical outcomes in CKD patients with asymptomatic ventricular arrhythmias. One of the most commonly used parameters reflecting renal function is creatine level and, in healthy individuals, it depends on the total muscle mass and the hydration status of the body (in dehydration states creatinine may increase) [5].

One of the most widespread cardiovascular risk factors is hypertension. Some studies on groups of healthy individuals have shown that systolic blood pressure becomes higher with advancing age, and that QTc significantly correlated with it [6].

On the other hand, SARS-CoV-2 infection is associated with a variety of pro-inflammatory mediators, such as IL-6 (interleukin-6), that may play important roles in the pathophysiology of cardiac and arrhythmic complications.

Most of the information about the prognostic role of the QT interval in SARS-CoV-2 infection has been derived from studies analyzing effects of the treatment with hydroxychloroquine and azithromycin, which are well known from QT interval prolongation. Fluorochinolons, especially levofloxacin, were widely used as first line antibiotics in patients with COVID-19. Its mechanism of action is to block hERG channels, causing a minor but significant prolongation of the QTc interval [7]. It is worth noting at this point, that levofloxacin is also a popular antibiotic widely used to treat bacterial infections of the lower respiratory tract.

On a 12-lead electrocardiography (ECG), the QT interval is measured from the beginning of the QRS complex to the end of the T wave as it returns to baseline. Manual measurements of the QT interval should be taken from leads II and V5 or V6 with the longest value being used. Measurements taken from these leads have the greatest positive and negative predictive value in detecting abnormal QT intervals [8]. Normal values for the QTc range from 350 to 450 ms for adult men and from 360 to 460 ms for adult women. In our analysis, we have assumed a cut-off value for QTc of 460 ms. There are numerous methodologies for correcting QT intervals for heart rate; however, there is no consensus as to determine which of them is the most effective. The most universally adopted method is Bazett’s formula (QTc = QT/√RR in seconds) that provides an adequate correction for heart rate, ranging anywhere between 60 and 100 beats/min [9]. The QT interval, discussed in this paper, is an indirect measure of the duration of ventricular depolarization and repolarization, and corresponds to the total duration of ventricular activation and recovery, the ventricular action potential. ECG patterns of ventricular repolarization may represent a potential tool for risk stratification.

The primary objective of this analysis was to assess the effect of levofloxacin on the corrected QT interval in COVID-19 patients. The results were compared to the control group not receiving this treatment. The secondary aim of this study was to show some trends for QT interval prolongation and identify the sociodemographic, clinical, and biochemical parameters associated with QTc prolongation in hospitalized patients with COVID-19. Given the increased risk of arrhythmias and adverse cardiovascular events linked to prolonged QTc in this population, understanding the factors that contribute to QTc variability is crucial to improving patient outcomes. The analysis aimed to explore these associations using both univariate and multivariate approaches, allowing for a comprehensive assessment of the independent effects of various clinical and demographic characteristics.

## 2. Materials and Methods

The medical records of 117 patients, hospitalized at the Department of Hypertension, Gastroenterology and Internal Medicine of the Medical University of Bialystok Clinical Hospital between November 2020 and April 2021, were retrospectively analyzed. The inclusion criteria for the study were a positive PCR test result, confirming COVID-19 infection, and elevated IL-6 and CRP (C-reactive protein) levels (as the main indicators of inflammation). Patients were including in the database, considering the parameters and comorbidities assessed at admission (Table 1). Patients who received other drugs that could potentially affect QT interval were excluded from the study.

Initially, every patient was hospitalized in the Department of Infectious Diseases for 3–5 days. At admission inflammatory markers (IL-6, CRP) were determined, and levofloxacin treatment was initiated (dose: 500 mg intravenous twice daily). A 12-lead ECG was performed on the day the patient was transferred to the Department of Hypertension, Gastroenterology and Internal Medicine (after 3–5 days of levofloxacin therapy). The QTc interval was calculated using the Bazzet formula [10]. Measurements of the QT interval were taken from leads II and V5 or V6 with the longest value being used. Other selected biochemical parameters, excluding CRP and IL-6, (listed in Table 2) were taken during hospitalization in the Department of Hypertension, Gastroenterology and Internal Medicine, using standard methods in the hospital laboratory, were obtained from the Clininet hospital system.

Records missing key parameters for statistical analysis were removed from the database, yielding a final group of 93 patients. Data from 93 adult patients were analyzed, with an age range of 39–94 years and a median age of 69 years. The male-to-female ratio in the cohort was 1:11, indicating a slight predominance of male patients. Then, we separated patients who did not receive levofloxacin—they constituted the control group. The analysis did not consider the effect of applied pharmacotherapy of chronic diseases (hypertension, diabetes or heart failure) on the QTc interval. Subsequently, selected biochemical and clinical parameters were subjected to statistical analysis, with particular emphasis on their effect on the QTc interval.

## 3. Statistical Analysis

Statistical analysis was conducted with a significance threshold of α = 0.05, corresponding to a 5% risk of Type I error (incorrect rejection of the null hypothesis). Descriptive statistics were used to summarize the data. Continuous variables were reported as medians (*Mdn*) along with the interquartile range (*IQR*), defined by the first (*Q1*) and third quartiles (*Q3*), which provided robust measures that are influenced less by outliers and skewed distributions. Categorical variables were summarized as frequencies (*n*) and percentages.

For comparisons between two independent groups, the Wilcoxon rank-sum test was employed for continuous variables that did not meet the assumption of normality. The associations between two numerical variables, without assuming normality, were evaluated using Spearman’s correlation, with the strength and direction of the relationship represented by Spearman’s Rho. Statistical significance was assessed using an asymptotic approximation of the *t*-test statistic.

The effect of multiple clinical parameters on QTc, a continuous outcome variable, was analyzed through a multivariate linear regression model. Model selection was performed using a stepwise procedure based on the Akaike Information Criterion (AIC) with backward elimination to optimize model fit while reducing dimensionality. The impact of each predictor on QTc was quantified by non-standardized regression coefficients (β), and model performance was evaluated through R-squared (R^2^) and adjusted R-squared (R^2^ adjusted), which reflect the explanatory power of the model.

Multicollinearity was assessed using the Variance Inflation Factor (VIF). A VIF greater than 10 typically indicates problematic multicollinearity, although values between 5 and 10 are also considered concerning in some cases. In this analysis, VIFs were monitored to ensure the stability and reliability of the regression coefficients, mitigating the risks of inflated variance due to correlations among predictors.

Based on the findings from Table 1 and Table 2, 11 potential covariates were identified for further validation in a multivariate model. These covariates, which include both clinical and laboratory parameters, demonstrated at least a trend-level association with QTc prolongation in the univariate analysis. This approach aims to identify the most relevant clinical determinants of QTc variability, providing more accurate risk stratification and guiding clinical decision-making in patients at risk for QTc-related complications. The application of a backward elimination approach successfully reduced the number of covariates to five. The results of the linear regression model in Table 3 provide important insights into the clinical factors associated with QTc prolongation.

## 4. Results

To explore the relationship between the QTc interval and various sociodemographic and clinical categorical parameters, a univariate analysis was conducted with results presented in Table 3. QTc values were compared across groups stratified by factors such as sex, smoking status, comorbidities (e.g., hypertension, diabetes mellitus, atrial fibrillation, heart failure), and COVID-19 severity. The characteristics and distributions of the sociodemographic and clinical parameters were reported in Table 1 and Table 2. All listed comorbidities were diagnosed prior to hospitalization for COVID-19. The severity of the COVID-19 course was based on the physician’s subjective assessment at the time of the admission (saturation less than 90%, resting dyspnoea, or additional respiratory muscles involvement).

### 4.1. Univariate Analysis

The receiving of levofloxacin, which was identified as a substance potentially affecting myocardial depolarization and repolarization, in our study did not reach statistical significance for its effect on QT interval. Also, markers of inflammation, including CRP and IL-6, did not show significant correlations with QTc.

In a univariate analysis based on the data in Table 1, it is shown that hypertension was significantly associated with longer QTc intervals, with patients exhibiting a median QTc (420 ms) compared to those without hypertension (385 ms) (*p* = 0.020). The history of paroxysmal atrial fibrillation (pAF) was also significantly associated with QTc prolongation, with patients having a median QTc of 438 ms compared to 415 ms in those without this arrhythmia (*p* = 0.018). Similarly, heart failure of any origin (with preserved, mildly reduced, and reduced ejection fraction) was associated with longer QTc intervals, (median QTc 430 ms compared to 410 ms) (*p* = 0.018).

We confirmed that heart rate (HR) demonstrated a negative correlation with QTc (Rho = −0.18, *p* = 0.078), approaching significance. This relationship is expected, as faster heart rates often shorten the QT interval, and this trend underscores the need to adjust QT measurements (i.e., QTc) for heart rate to provide more reliable assessments across different clinical scenarios.

There was a near-significant trend observed in the relationship between COVID-19 severity and QTc prolongation, with patients who experienced moderate to severe COVID-19 course (median QTc 430 ms) compared to those with mild disease (median QTc 400 ms) (*p* = 0.054). While this finding did not reach statistical significance, the trend suggests that more severe COVID-19 may contribute to QTc prolongation.

Potassium level showed a negative correlation with QTc (Rho = −0.19, *p* = 0.073), approaching significance. Given the well-established association between hypokalemia and QTc interval prolongation.

Among other studied parameters, age was found to be the most significant factor, showing a moderate positive correlation with QTc (Rho = 0.34, *p* < 0.001). On the other hand, in multivariate analysis, the effect of age on the QTc interval did not reach statistical significance, indicating that in elderly patients the influence of other factors, such as concomitant diseases and pharmacotherapy, has a more significant impact on QT length than age per se.

**Figure 1:**
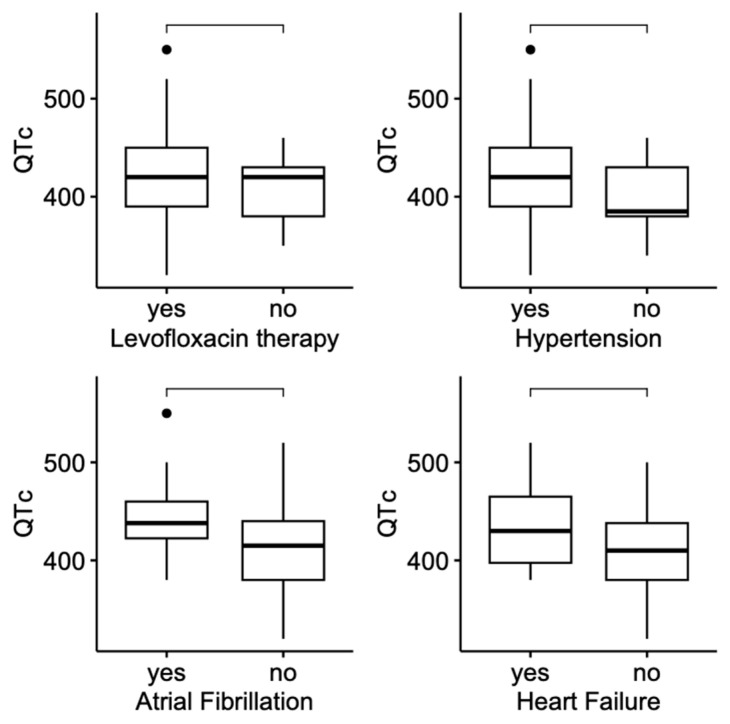
The distribution of QTc values in patient groups where statistical significance was achieved (α = 0.05).

To further investigate potential determinants of QTc prolongation, correlations between QTc and continuous sociodemographic and clinical parameters were examined. This analysis provides insight into the relationships between QTc and variables such as age, body composition, vital signs, inflammatory markers, and laboratory findings. The strength and direction of those correlations, along with their statistical significance, are presented in Table 4. The distribution of QTc values in patient groups where statistical significance was achieved is shown in Figure 1.

### 4.2. Multivariate Analysis

Multivariate analysis for identifying factors associated with QTc and the results of the linear regression model (presented in Table 5) provided important insights into the clinical factors associated with QTc prolongation. Covariates were chosen due to their potential influence on QTc variability, as suggested by their individual associations or near-significant trends. The interception of β_0_ = 391.67 ms represents the baseline QTc value for a patient with median values of heart rate (80 beats/min), creatinine (0.93 mg/dL), and eGFR (75.0 mL/min/1.73 m^2)^, without hypertension or heart failure.

Creatinine level was significantly associated with QTc prolongation (β = 38.77 ms, *p* = 0.031). For every 1.0 mg/dL increase in creatinine concentration above the median (0.93 mg/dL), QTc increased by approximately 39 ms, indicating that impaired renal function is a significant predictor of QTc prolongation.

Hypertension showed a borderline association with QTc prolongation (β = 18.11 ms, *p* = 0.088), suggesting that patients with hypertension may have a longer QTc compared to those without this condition, though this did not reach statistical significance.

The results of univariate analysis confirmed that heart failure was significantly associated with QTc prolongation (β = 24.07 ms, *p* = 0.027). These patients had QTc intervals that were on average 24 ms longer than those without heart failure.

### 4.3. Nonparametric Statistical Analysis

Patients treated with levofloxacin (Table S1 in the Supplementary Materials) presented a more severe disease course, with a higher incidence of moderate (53.8% vs. 13.3%) and severe (12.8% vs. 0%) cases compared to those not receiving levofloxacin (*p* = 0.001), along with lower oxygen saturation (89.0% vs. 91.0%, *p* = 0.038) and elevated ferritin levels (940.8 vs. 648.8 µg/L, *p* = 0.015), confirming its use in critically ill patients with obvious inflammation.

In contrast, patients with prolonged QTc (Table S2) were older (76.0 vs. 68.5 years, *p* = 0.036), had a higher prevalence of heart failure (43.8% vs. 18.3%, *p* = 0.046) and had higher diastolic blood pressure (82.0 vs. 73.0 mmHg, *p* = 0.011), suggesting a cardiac risk profile that predisposes to arrhythmias.

Elevated ferritin and C-reactive protein, appeared to be common in both groups, although only ferritin was significant in the levofloxacin cohort and lymphocyte counts were significantly lower in levofloxacin-treated patients (0.9 vs. 1.4 × 10^3^/µL, *p* = 0.013, Table S1).

While levofloxacin is a known QTc-prolonging agent, no direct association with QTc risk factors like electrolyte imbalances was evident (e.g., potassium, *p* = 0.913, Table S1), demonstrating that age and heart failure are stronger drivers of QTc prolongation (Table S2 in the Supplementary Materials).

## 5. Discussion

Results of our retrospective study showed that levofloxacin, an antibiotic commonly used during the SARS-CoV2 pandemic and one of the most commonly used fluoroquinolones, showed no association with QTc interval prolongation. The group of patients we studied was heterogeneous and characterized by a significant burden of comorbidities: 20% of patients presented with heart failure, 78% were diagnosed with hypertension, 36% had type 2 diabetes, and 18% had paroxysmal atrial fibrillation. The above-mentioned presence of multiple comorbidities could have affected the pharmacokinetics and pharmacodynamics of levofloxacin by, for example, impairing its absorption. Levofloxacin is a lipophilic drug, so in people who are obese its activity may be altered, both in the direction of decreasing the effect, as well as prolonging or delaying the effect. In our group of patients the mean BMI in the study group was 27.49 kg/m^2^ (distribution: 24.55, 32.31). Also, in our study, the group of patients not receiving levofloxacin was small (*n* = 15), so that the power of the test was reduced and the risk of type II error was increased (false negative result). This means that it is very likely that the true effect of levofloxacin on the Qtc interval may not be detected due to the low “power” of the test. Despite this, the effect of levofloxacin on the QT interval reported in the literature is inconclusive. Kervezee [11] et al. performed a study on the group of 12 healthy male subjects, indicating a dose-dependent prolongation of the QTc interval and a diurnal fluctuation in serum drug concentration. Drug-induced long QT syndrome is implicated by a blockade of cardiac voltage-gated potassium channels, particularly the rapid component (IKr) of the delayed rectifier potassium current (IK), expressed by HERG [12]. By doing so, fluoroquinolones usually, but not always, prolong the QT interval. A recent trial demonstrated that levofloxacin had little affinity to this channel [13]. QT interval prolongation predisposes patients to ventricular arrhythmias such as torsade de pointes (TdP). In the U.S., data reported to the pharmaceutical institutes, shows TdP at a rate of 1 per million levofloxacin prescriptions, similar to the TdP rate associated with ciprofloxacin. Our finding underlines how important it is to take caution during the use of levofloxacin antibiotic therapy in patients with chronic kidney disease, which often leads to electrolyte disturbances.

Our study concludes that markers of systemic inflammation (IL-6, CRP) may not be the main driver of QTc interval prolongation, despite its known association with cardiovascular risk. However, it is important to consider that these markers may still affect QTc in specific subgroups of patients, particularly those with sepsis-induced myocardial damage in septic shock or systemic inflammatory response syndrome [14]. To emphasize the physiological response of an infection, it is also worth mentioning that, an animal model study found that serum concentrations of IL-6 increased and peaked at 12 h. The terminal elimination half-life of IL-6 was 15.5 h. The results demonstrated that these cytokines synthesized in response to inflammatory stimulation were rapidly eliminated [15]. Thus, the probability of a false-negative result, probably increased the time between the measurement of Il-6 and CRP and the time the ECG was performed. At this point, it is worth mentioning again that the study group was heterogeneous, so that the timing of IL-6 elimination could be variable. The estimated diurnal variability is large enough to require IL-6 sampling at a consistent time of the day [16].

The univariate analysis and complex multivariate analysis showed that hypertension, as an independent factor, significantly affects prolongation of the corrected QT interval, potentially increasing the risk of arrhythmias [17]. Even small, non-specific abnormalities of repolarization may increase the risk of cardiovascular events in the hypertensive population [18]. Also, QTc tends to increase with age, possibly due to age-related changes in cardiac electrophysiology and structural heart disease, which is consistent with the existing literature [19]. This underscores the importance of age as a key factor in stratifying the risk of QTc interval prolongation in older patients when selecting pharmacological treatment.

We discovered that patients with a history of paroxysmal atrial fibrillation also tended to have a prolonged QTc, compared to patients without a history of this arrhythmia. All findings were confirmed by research showing that prolonged QT interval predicts an approximately twofold increased risk of atrial fibrillation [20,21]. Those results suggest that ventricular repolarization is an easily identifiable risk marker for atrial fibrillation that may shed new light on the pathophysiology of this arrhythmia. Furthermore, Kario [22] et al. demonstrated that poor control of blood pressure in elderly patients is associated with a higher incidence of new-onset atrial fibrillation and poorer cardiovascular outcomes of atrial fibrillation compared with normotensive or well-controlled hypertensive patients. One may therefore venture to conclude that in an exemplary elderly patient, hospitalized for an infectious disease and treated for hypertension and paroxysmal atrial fibrillation, the risk of QT prolongation warrants attention and monitoring.

In addition, heart failure of any origin was associated with prolongation of the QTc interval. It further highlights the need for close monitoring of these patients during the use of drugs that affect myocardial repolarization [23]. The prolongation of the QTc interval in this context explains the increased susceptibility to malignant ventricular arrhythmias, which is often associated with implantation of a cardioverter defibrillator (ICD) for secondary prevention in this patient group [24]. Among patients with heart failure taking oral diuretics, particularly those at risk of developing hypokalemia, this trend, which presents a negative correlation between potassium levels and QTc, is clinically relevant [25]. It suggests that even in the normal range, lower potassium levels may contribute to QTc interval prolongation.

In our study among patients with SARS-CoV2 infection and cardio-vascular risk factors, a multivariate analysis revealed that an increase in creatinine concentration had a direct effect on QT prolongation. This is a very important finding in understanding the risk factors for dangerous ventricular arrhythmias in patients with increased creatinine levels who do not meet the diagnostic criteria for chronic kidney disease. It was shown that for every 1.0 mg/dL increase in creatinine above the median (0.93 mg/dL), QTc increased by approximately 39 ms. These results clearly indicate a strong effect of creatinine concentration on QTc. This means that patients’ renal function should be monitored before initiating treatment to prolong the QT interval. Especially in older patients who are easily dehydrated and have water-electrolyte imbalances [26]. This finding highlights the need for heightened vigilance in patients with renal dysfunction, as they may be at increased risk of arrhythmias due to prolonged QTc.

## 6. Conclusions

In summary, our retrospective study showed prolongation of the corrected QT interval as a simultaneous occurrence of multiple factors. It should not be assumed that only the use of a drug with a known effect on QTc interval can predispose to the occurrence of QT-prolongation-dependent ventricular arrhythmias. Considering the results, regular monitoring of the ECG should be performed in elderly patients with increased creatinine levels and heart failure of any origin. Extra caution, before initiating treatment affecting the QT interval, should be maintained in patients with heart failure who have the “cold and dry” phenotype of heart failure. This heart failure phenotype is often associated with septic disorders, and is characterized by dehydration and electrolyte imbalances, and associated with a poor prognosis [27]. It is reasonable to presume that serum creatinine concentration may be an easily assessable predictor of QTc interval prolongation. Given that hypertension is the most widespread cardiovascular risk factor worldwide, and atrial fibrillation is the most prevalent clinically significant rhythm disorder in the elderly, these patients should have a 12-lead ECG with QT interval assessment on the first day of hospitalization. It is a very affordable and easy to perform examination that can expose potential risks of arrhythmias that we can prevent. It is also worth considering the use of telehealth technologies, including artificial intelligence, to monitor patients who require a more attentive approach [28].

## 7. Study Limitations

Our study has several limitations that must be taken into account. First, the study group was relatively small, which may limit the generalizability of the results, especially small sample sizes in nonparametric statistical analysis (N = 15 for non-levofloxacin, N = 19 for prolonged QTc) and missing data warrant caution in generalizing these results, and further research is needed to clarify levofloxacin’s specific contribution to QTc prolongation.

Second, the study was conducted in a single center, which potentially introduces selection bias. Third, as a retrospective study, it cannot establish a causal relationship between levofloxacin use and QT interval prolongation, only an association (statistical relationship) between variables. In addition, the data came from medical records, which may lead to incomplete or inaccurate information. Despite these limitations, our results provide valuable insights, and future studies with larger, more diverse populations and prospective designs are needed to confirm our findings.

## Figures and Tables

**Table 1:** Characteristics and distributions of the sociodemographic parameters. Note: *n_pairs_*—number of pairs of parameters.

Parameter	Group	*n*
Levofloxacin therapy	Yes	78
	No (control group)	15
Sex	Female	44
	Male	49
Smoking	Yes	11
	No	79
Hypertension	Yes	73
	No	20
Diabetes Mellitus	Yes	33
	No	59
Atrial Fibrillation (paroxysmal)	Yes	16
	No	74
Heart Failure	Yes	20
	No	67
COVID-19 severity	Mild	39
	Moderate/Severe	54
Resting dyspnea	Yes	47
	No	46

**Table 2:** Characteristics and distributions of clinical parameters.

Parameter	*n_pairs_*	Distribution ^a^
Il-6, pg/mL	69	73.20 (40.80, 130.80)
CRP, mg/L	92	84.32 (36.60, 141.54)
BMI, kg/m^2^	66	27.89 (24.55, 32.31)
Patient’s age, years	93	69.00 (60.00, 81.00)
Saturation, %	87	90.00 (83.50, 93.00)
SBP, mmHg	93	120.00 (110.00, 134.00)
DBP, mmHg	93	75.00 (67.00, 81.00)
HR, beats/min	92	80.00 (71.00, 94.25)
ALT, μ/L	85	37.00 (22.00, 64.00)
AST, μ/L	75	30.00 (22.50, 46.50)
WBC, 10^3^/μL	92	7.08 (5.13, 9.27)
NEU, 10^3^/μL	90	4.98 (3.35, 7.27)
LYM, 10^3^/μL	89	0.98 (0.61, 1.36)
HGB, g/dL	92	13.00 (11.60, 14.13)
MCV, fL	92	86.30 (82.78, 90.13)
PLT, 10^3^/μL	92	179.50 (144.74, 291.50)
Creatinine, mg/dL	89	0.93 (0.79, 1.17)
eGFR, mL/min/1.73 m^2^	89	75.00 (60.10, 95.90)
Potassium, mmol/L	93	4.54 (3.97, 5.01)
Sodium, mmol/L	92	139.00 (136.00, 141.00)
Ferritin, μg/L	66	876.10 (541.35, 1478.00)
Fibrinogen, mg/dL	81	527.00 (364.00, 663.00)
D-dimers. mg/L FEU	88	1298.50 (682.25, 2143.50)

Note: *n_pairs_*—number of pairs of parameters. ^a^ *Mdn (Q1, Q3)*; BMI, body mass index; CRP, c-reactive protein; DBP, diastolic blood pressure; eGFR, estimated glomerular filtration rate; HR, heart rate; SBP, systolic blood pressure.

**Table 3:** Distribution of QTc across groups stratified by categorical sociodemographic and clinical factors in the study sample, with estimation of statistical significance of between-group differences. ^a^ *Mdn (Q1, Q3)*; ^b^ Wilcoxon sum rank test; Note: *n*–group size; *p*—*p*-value of the statistical test; *Mdn*—median; *Q1*—the first quartile (25%); *Q3*—the third quartile (75%).

Parameter	Group	*n*	QTc Distribution ^a^	*p* ^b^
Levofloxacin therapy	Yes	78	420.00 (390.00, 450.00)	0.293
	No (control group)	15	420.00 (380.00, 430.00)	
Sex	Female	44	420.00 (380.00, 446.25)	0.700
	Male	49	420.00 (390.00, 440.00)	
Smoking	Yes	11	410.00 (362.50, 420.00)	0.120
	No	79	420.00 (385.00, 450.00)	
Hypertension	Yes	73	420.00 (390.00, 450.00)	**0.020**
	No	20	385.00 (380.00, 430.00)	
Diabetes Mellitus	Yes	33	410.00 (385.00, 440.00)	0.590
	No	59	420.00 (385.00, 447.50)	
Atrial Fibrillation (paroxysmal or persistent)	Yes	16	438.00 (422.50, 460.00)	**0.018**
	No	74	415.00 (380.00, 440.00)	
Heart Failure	Yes	20	430.00 (397.50, 465.00)	**0.018**
	No	67	410.00 (380.00, 438.00)	
COVID-19 severity	Mild	39	400.00 (380.00, 433.00)	0.054
	Moderate/Severe	54	430.00 (390.00, 450.00)	
Resting dyspnea	Yes	47	420.00 (380.00, 450.00)	0.732
	No	46	420.00 (390.00, 440.00)	

**Table 4:** Correlations between QTc, sociodemographic and clinical parameters with their statistical significance.

Parameter	*n_pairs_*	Distribution ^a^	QTc, 420.00 (385.00, 445.00) ms		
			Rho	CI 95%	*p*
**Il-6, pg/mL**	**69**	73.20 (40.80, 130.80)	−0.05	−0.29–0.20	0.698
CRP, mg/L	92	84.32 (36.60, 141.54)	−0.08	−0.29–0.13	0.451
BMI, kg/m^2^	66	27.89 (24.55, 32.31)	−0.03	−0.28–0.22	0.796
Patient’s age, years	93	69.00 (60.00, 81.00)	0.34	0.14–0.52	**<0.001**
Saturation, %	87	90.00 (83.50, 93.00)	0.08	−0.14–0.29	0.479
SBP, mmHg	93	120.00 (110.00, 134.00)	0.11	−0,10–0,31	0.306
DBP, mmHg	93	75.00 (67.00, 81.00)	0.16	−0.05–0.36	0.116
HR, beats/min	92	80.00 (71.00, 94.25)	−0.18	−0.38–0.03	0.078
ALT, μ/L	85	37.00 (22.00, 64.00)	−0.04	−0.26–0.18	0.717
AST, μ/L	75	30.00 (22.50, 46.50)	0.02	−0.21–0.25	0.849
WBC, 10^3^/μL	92	7.08 (5.13, 9.27)	−0.05	−0.26–0.16	0.609
NEU, 10^3^/μL	90	4.98 (3.35, 7.27)	−0.07	−0.28–0.15	0.518
LYM, 10^3^/μL	89	0.98 (0.61, 1.36)	−0.12	−0.33–0.09	0.252
HGB, g/dL	92	13.00 (11.60, 14.13)	−0.07	−0.28–0.14	0.478
MCV, fL	92	86.30 (82.78, 90.13)	0.20	−0.01–0.40	0.053
PLT, 10^3^/μL	92	179.50 (144.74, 291.50)	−0.16	−0.36–0.05	0.120
Creatinine, mg/dL	89	0.93 (0.79, 1.17)	0.18	−0.03–0.38	0.090
eGFR, mL/min/1.73 m^2^	89	75.00 (60.10, 95.90)	−0.15	−0.35–0.06	0.155
Potassium, mmol/L	93	4.54 (3.97, 5.01)	−0.19	−0.38–0.02	0.073
Sodium, mmol/L	92	139.00 (136.00, 141.00)	0.03	−0.18–0.24	0.793
Ferritin, μg/L	66	876.10 (541.35, 1478.00)	0.16	−0.10–0.39	0.209
Fibrinogen, mg/dL	81	527.00 (364.00, 663.00)	−0.08	−0.30–0.15	0.486
D-dimers. mg/L FEU	88	1298.50 (682.25, 2143.50)	0.12	−0.09–0.33	0.250

^a^ *Mdn (Q1, Q3)*, Note: *n_pairs_*—number of pairs of parameters, Rho—regression coefficient estimated by the Spearman method, CI 95%—confidence interval 95%; *p*—*p*-value of the statistical test; BMI, body mass index; CRP, c-reactive protein; DBP, diastolic blood pressure; eGFR, estimated glomerular filtration rate; HR, heart rate; SBP, systolic blood pressure.

**Table 5:** Multivariate analysis for identifying factors associated with QTc and the results of the linear regression model. Note: β—regression coefficient (non-standardized); CI 95%—confidence interval 95%; *p*—*p*-value of t Student test.

	QTc, ms		
Predictors	*β*	CI 95%	*p*
(Intercept)	−0.05	−0.29–0.20	0.698
HR (centered by the *Mdn* = 80 beats/min)	−0.08	−0.29–0.13	0.451
Creatinine (centered by the *Mdn* = 0.93 mg/dL)	−0.03	−0.28–0.22	0.796
eGFR (centered by the *Mdn* = 75.0 mL/min/1.73 m^2^)	0.34	0.14–0.52	**<0.001**
**Hypertension**			
No	Reference level		
Yes	18.11	−2.79–39.02	0.088
**HF LVEF =< 60%**	Reference level		
No			
Yes	24.07	2.78–45.35	**0.027**

eGFR, estimated glomerular filtration rate; HR, heart rate; HF, heart failure; LVEF, left ventricular ejection fraction.

## Data Availability

Data are contained within the article.

## Supplementary Materials

The following supporting information can be downloaded at: https://www.mdpi.com/article/10.3390/jcm14114006/s1, Table S1: Baseline characteristics of patients stratified by Levofloxacin therapy.; Table S2: Baseline characteristics of patients stratified by prolonged QTc interval.
