# Supplementary material for: Heart Failure, Kidney Function, and Elderly Age, Rather than Levofloxacin Therapy, Are Associated with QTc Prolongation in COVID-19 Patients
Source: J Clin Med. 2025 Jun 5;14(11):4006. doi: 10.3390/jcm14114006 (PMC12156308; doi:10.3390/jcm14114006)
Supplement: Supplementary file 1 [file jcm-14-04006-s001.zip › jcm-3682477-supplementary.pdf]

Supplements:

Table S1. Baseline characteristics of patients stratified by Levofloxacin therapy

**Characteristic**	**N**	**Levofloxacin therapy (N = 78)**	**No levofloxacin therapy (N = 15)**	**p**
**Demographics,**				
Sex, n (%)	93			0.282
Female		35 (44.9%)	9 (60.0%)	
Male		43 (55.1%)	6 (40.0%)	
Age, years, median (IQR)	93	70.5 (60.3, 81.0)	69.0 (62.0, 75.0)	0.711
Body Mass Index, kg/m², median (IQR)	66	27.4 (24.2, 32.3)	29.7 (27.9, 32.5)	0.132
Current Smoker, n (%)	90	7 (9.3%)	4 (26.7%)	0.082
**Clinical Characteristics, n (%)**				
Hypertension	93	61 (78.2%)	12 (80.0%)	1.000
Diabetes Mellitus	92	28 (36.4%)	5 (33.3%)	0.823
Atrial Fibrillation	90	11 (14.5%)	5 (35.7%)	0.120
Heart Failure (LVEF <60%)	87	16 (21.9%)	4 (28.6%)	0.729
Disease Severity:	93			**0.001**
Mild		26 (33.3%)	13 (86.7%)	**< 0.001**
Moderate		42 (53.8%)	2 (13.3%)	**0.004**
Severe		10 (12.8%)	0 (0.0%)	0.356

Characteristic	N	Levofloxacin therapy (N = 78)	No levofloxacin therapy (N = 15)	p
Dyspnea	93	41 (52.6%)	6 (40.0%)	0.373
Oxygen Saturation (without O_2), %, median (IQR)	87	89.0 (82.0, 93.0)	91.0 (89.3, 94.8)	**0.038**
Systolic Blood Pressure, mmHg, median (IQR)	93	119.5 (110.0, 133.0)	120.0 (112.5, 135.5)	0.397
Diastolic Blood Pressure, mmHg, median (IQR)	93	74.5 (67.0, 80.0)	75.0 (69.0, 86.5)	0.699
Heart Rate, beats/min, median (IQR)	92	80.0 (71.0, 94.0)	80.0 (71.0, 94.5)	0.937
**Laboratory Parameters, median (IQR)**				
IL-6, pg/mL	69	73.2 (40.8, 140.4)	79.6 (51.9, 97.0)	0.740
C-Reactive Protein, mg/L	92	86.0 (46.1, 142.3)	40.8 (26.6, 94.1)	0.202
Alanine Aminotransferase, U/L	85	37.0 (22.3, 63.3)	34.0 (19.0, 68.0)	0.628
Aspartate Aminotransferase, U/L	75	30.0 (22.8, 46.3)	34.0 (25.5, 45.5)	0.701
White Blood Cell Count, $\times 10^3/\mu L$	92	7.9 (5.2, 9.6)	6.3 (4.5, 7.8)	0.258
Neutrophil Count, $\times 10^3/\mu L$	90	5.2 (3.6, 7.4)	3.4 (3.0, 5.3)	0.076
Lymphocyte Count, $\times 10^3/\mu L$	89	0.9 (0.6, 1.3)	1.4 (0.9, 1.5)	**0.013**
Hemoglobin, g/dL	92	13.0 (11.9, 14.2)	12.8 (10.8, 13.9)	0.308
Mean Corpuscular Volume, fL	92	86.2 (82.5, 90.1)	86.5 (84.6, 89.9)	0.401

Characteristic	N	Levofloxacin therapy (N = 78)	No levofloxacin therapy (N = 15)	p
Platelet Count, $\times 10^3/\mu L$	92	176.0 (143.0, 291.0)	209.0 (167.5, 305.5)	0.265
Creatinine, mg/dL	89	0.95 (0.79, 1.21)	0.82 (0.78, 0.91)	0.100
eGFR, mL/min/1.73 m^2	89	74.8 (58.4, 95.1)	81.8 (73.2, 99.2)	0.262
Potassium, mmol/L	93	4.55 (3.95, 5.01)	4.42 (4.13, 4.97)	0.913
Sodium, mmol/L	92	139.0 (136.0, 141.0)	139.0 (137.0, 140.5)	0.836
Ferritin, μg/L	66	940.8 (580.9, 1,551.8)	648.8 (374.7, 742.1)	**0.015**
Fibrinogen, mg/dL	81	539.5 (385.8, 681.0)	403.0 (339.0, 501.0)	0.063
D-Dimer, μg/L FEU	88	1,323.0 (751.5, 2,031.8)	1,156.0 (489.5, 3,641.5)	0.780

Notes: Data are presented as n (%) for categorical variables and median (interquartile range, IQR) for continuous variables. p-Values derived from Pearson's Chi-squared test for categorical variables, Wilcoxon rank-sum test for continuous variables, or Fisher's exact test where appropriate. Abbreviations: LVEF, left ventricular ejection fraction; IL-6, interleukin-6; eGFR, estimated glomerular filtration rate; FEU, fibrinogen equivalent units. Sample sizes vary due to missing data, as reflected in the "N" column.

Table S2. Baseline characteristics of patients stratified by prolonged QTc interval

Characteristic	N	Prolonged QTc (N = 19)	Non-Prolonged QTc (N = 74)	p
**Demographics**				
Sex, n (%)	93			0.306
Female		7 (36.8%)	37 (50.0%)	
Male		12 (63.2%)	37 (50.0%)	
Age, years, median (IQR)	93	76.0 (66.0, 86.0)	68.5 (59.0, 79.5)	**0.036**
Body Mass Index, kg/m², median (IQR)	66	28.4 (26.8, 31.8)	27.6 (24.4, 34.2)	0.955
Current Smoker, n (%)	90	0 (0.0%)	11 (15.3%)	0.112
**Clinical Characteristics, n (%)**				
Hypertension	93	18 (94.7%)	55 (74.3%)	0.064
Diabetes Mellitus	92	6 (31.6%)	27 (37.0%)	0.662
Atrial Fibrillation	90	5 (26.3%)	11 (15.5%)	0.315
Heart Failure (LVEF <60%)	87	7 (43.8%)	13 (18.3%)	**0.046**
Disease Severity	93			0.269
Mild		5 (26.3%)	34 (45.9%)	
Moderate		11 (57.9%)	33 (44.6%)	
Severe		3 (15.8%)	7 (9.5%)	
Dyspnea	93	12 (63.2%)	35 (47.3%)	0.217

Characteristic	N	Prolonged QTc (N = 19)	Non-Prolonged QTc (N = 74)	p
Oxygen Saturation (without O_2), %, median (IQR)	87	86.0 (81.5, 93.0)	90.0 (84.0, 93.0)	0.481
Systolic Blood Pressure, mmHg, median (IQR)	93	123.0 (118.0, 139.0)	117.5 (109.3, 131.0)	0.055
Diastolic Blood Pressure, mmHg, median (IQR)	93	82.0 (71.5, 86.5)	73.0 (66.0, 79.0)	**0.011**
Heart Rate, beats/min, median (IQR)	92	76.0 (67.0, 88.0)	80.0 (72.0, 95.0)	0.178
**Laboratory Parameters, median (IQR)**				
IL-6, pg/mL	69	77.1 (50.0, 123.1)	73.2 (40.8, 133.3)	0.823
C-Reactive Protein, mg/L	92	83.7 (36.8, 137.7)	84.9 (36.9, 141.7)	0.836
Alanine Aminotransferase, U/L	85	37.0 (23.0, 61.5)	36.5 (22.0, 63.3)	0.840
Aspartate Aminotransferase, U/L	75	32.5 (28.0, 43.0)	30.0 (22.0, 47.0)	0.712
White Blood Cell Count, $\times 10^3/\mu L$	92	6.9 (5.1, 8.5)	7.4 (5.2, 9.8)	0.493
Neutrophil Count, $\times 10^3/\mu L$	90	4.7 (3.6, 6.7)	5.0 (3.3, 7.3)	0.579
Lymphocyte Count, $\times 10^3/\mu L$	89	1.0 (0.6, 1.1)	1.0 (0.6, 1.4)	0.224
Hemoglobin, g/dL	92	12.5 (10.9, 13.9)	13.0 (11.9, 14.2)	0.337
Mean Corpuscular Volume, fL	92	86.4 (82.7, 90.7)	86.2 (83.0, 89.2)	0.619
Platelet Count, $\times 10^3/\mu L$	92	173.0 (124.5, 266.0)	187.0 (158.0, 293.0)	0.190

Characteristic	N	Prolonged QTc (N = 19)	Non-Prolonged QTc (N = 74)	p
Creatinine, mg/dL	89	1.04 (0.85, 1.90)	0.91 (0.79, 1.11)	0.124
eGFR, mL/min/1.73 m²	89	68.3 (35.3, 94.4)	77.6 (61.9, 95.4)	0.274
Potassium, mmol/L	93	4.22 (3.89, 4.87)	4.59 (4.08, 5.01)	0.232
Sodium, mmol/L	92	140.0 (136.0, 141.0)	139.0 (137.0, 141.0)	0.801
Ferritin, µg/L	66	985.3 (456.3, 1,267.0)	876.1 (566.3, 1,495.8)	0.761
Fibrinogen, mg/dL	81	477.0 (405.0, 568.0)	532.5 (364.0, 663.0)	0.697
D-Dimer, µg/L FEU	88	1,387.0 (795.0, 1,993.5)	1,294.0 (632.0, 2,160.0)	0.711

Notes: Data are presented as n (%) for categorical variables and median (interquartile range, IQR) for continuous variables. Prolonged QTc defined as ≥460 ms for females and ≥450 ms for males. The "N" column indicates the number of patients with available data for each characteristic. p-Values derived from Pearson's Chi-squared test for categorical variables, Wilcoxon rank-sum test for continuous variables, or Fisher's exact test where appropriate. Abbreviations: LVEF, left ventricular ejection fraction; IL-6, interleukin-6; eGFR, estimated glomerular filtration rate; FEU, fibrinogen equivalent units. Sample sizes vary due to missing data, as reflected in the "N" column.
